# Evolution in Environmental Health: Incorporating the Infectious Disease Paradigm

**DOI:** 10.1289/ehp.1002661

**Published:** 2010-08

**Authors:** Linda S. Birnbaum, Paul Jung

**Affiliations:** E-mail: birnbaumls@niehs.nih.gov; National Institutes of Health, Department of Health and Human Services, Research Triangle Park, North Carolina

In this issue of *Environmental Health Perspectives*, Feingold et al. (2010) propose a unique step forward for toxicology: incorporating infectious disease agents and theory into the toxicological paradigm.

The fields of infectious disease and toxicology intersect on many different levels. First, they can act concurrently, as when global bands of various tropical diseases widen due to increased atmospheric temperatures. For example, in *A Human Health Perspective on Climate Change,* the Interagency Working Group on Climate Change and Health (2010) identified health effects from climate change, as well as the health benefits from mitigating climate change. These various health effects range from respiratory and cardiovascular disease, to developmental and neurological disorders, to food- and waterborne illness, and vectorborne and zoonotic disease. It is increasingly clear that climate change—a marquee issue in the field of environmental health—and infectious disease are linked.

Second, the two fields can also act antagonistically: For example, the newly renewed appeals for global use of DDT (dichlorodiphenyltrichloroethane) to combat malaria will pit the well-known hazardous effects of DDT against the scourge of malaria. In many countries DDT has been banned for agricultural use; it is considered a Class II or “moderately hazardous” pesticide by the World Health Organization (International Programme on Chemical Safety 2005), and its use is strictly limited by the 2001 Stockholm Convention. However, use of DDT is still permitted for vector control. This balance of risks and benefits is a conundrum for scientists and policy makers, but it reveals the serious issues raised when infectious disease and environmental health interests clash.

Third, these two disciplines can act synergistically, as in the interactions between hepatitis B and aflatoxin in hepatic cancer. Both hepatitis B and aflatoxin are independent factors in liver cancer. However, when combined, they act powerfully to raise the risk of hepatic cancer up to 60 times that of unexposed individuals (Groopman et al. 2005). This National Institute of Environmental Health Sciences (NIEHS)-funded research is a primary example of the interaction between environmental health and infectious disease and can serve as a model for future research efforts.

Supression of the immune response by polychlorinated biphenyls (PCBs) was first shown in mice and nonhuman primates. Recently, in another example of concurrent interaction, NIEHS-funded studies led by Philippe Grandjean have shown that perinatal and developmental exposure to PCBs adversely impact immune responses to childhood vaccinations (Heilmann et al. 2006, 2010).

We have an opportunity at the NIEHS to embrace this new paradigm. As we have shown with our investment in research into the aflatoxin–hepatitis B and PCB–vaccine interactions, the NIEHS has a track record that could promote a wider interest in this field of inquiry.

Ideas like these are supported not only at the institute level but also throughout the National Institutes of Health (NIH). Recently, NIH director Francis Collins (2010b) wrote, “NIH can play a major role in ramping up the discovery of novel targets in both pathogen and host and work to facilitate advances in prevention . . . .” Collins (2010a) also wrote, “the best outcomes are generally when you don’t have walls between parts of the organization that prevent people from learning from each other.”

A recent presentation at the NIEHS outlined a vision for the institute that included the infectious disease and environmental health intersection within the context of the rapid evolution in the field of environmental health, specifically in epigenetics. As we recognize that our old assumptions about toxicants and how they affect our bodies are being changed by modern science (e.g., exposure effects are not only dose dependent but are also affected by both time and context), the field of environmental health is moving fast and the NIEHS needs to be at the front with innovative, bold ideas so we can participate and lead with the best science possible. The idea of incorporating infectious disease into the toxicological paradigm is exactly the kind of pioneering concept that can take environmental health to the next level.

The NIEHS Office of the Director will be working with division leaders to develop an initiative on infectious disease and environmental health—to incorporate infectious disease into the toxicological paradigm. We look forward to the possibilities to strengthen the field of environmental health science.

## Figures and Tables

**Figure:**
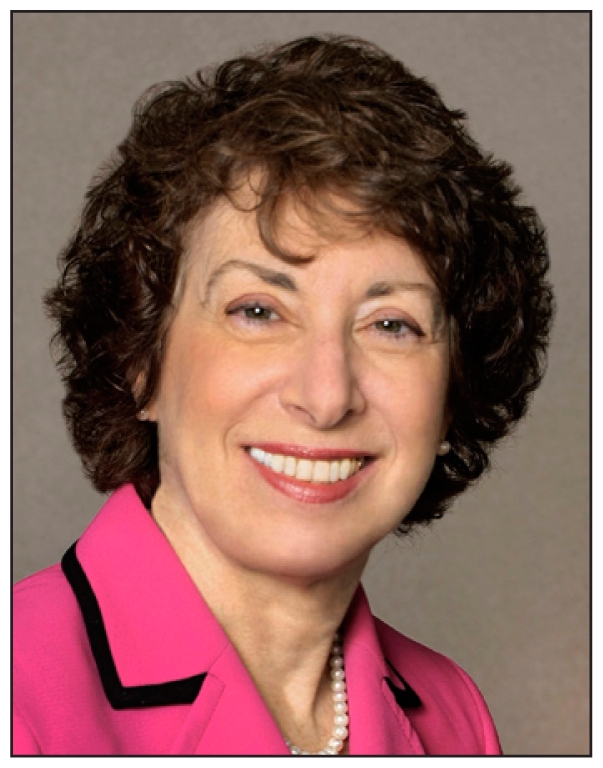
Linda S. Birnbaum

**Figure:**
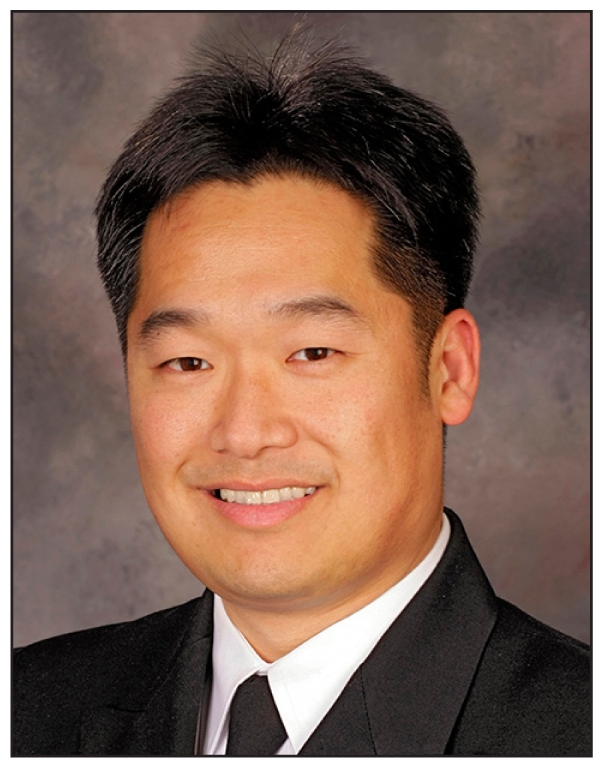
Paul Jung
